# Beyond the tailpipe: Review of non-exhaust airborne nanoparticles from road vehicles

**DOI:** 10.1016/j.eehl.2024.11.003

**Published:** 2024-12-16

**Authors:** Yingyue Wei, Prashant Kumar

**Affiliations:** a Global Centre for Clean Air Research (GCARE), School of Engineering, Civil and Environmental Engineering, Faculty of Engineering and Physical Sciences, University of Surrey, Guildford GU2 7XH, United Kingdom; b Institute for Sustainability, University of Surrey, Guildford GU2 7XH, United Kingdom

**Keywords:** PM_0.1_, Non-exhaust particles, Brake wear, Tyre wear, Air pollution

## Abstract

With the electrification of road vehicles leading to a reduction in tailpipe emissions, the relative contribution of non-exhaust emissions (NEEs) has become increasingly prominent. NEEs, particularly nanoparticles smaller than 100 nm in aerodynamic diameter (PM_0.1_), present significant health and environmental risks. A comprehensive understanding and strategic management of these emissions are urgently required to mitigate their impact. This article reviews existing studies and reveals that nanoparticles in NEEs are generated from brake and tyre wear under critical temperature conditions, while road wear and resuspension do not directly produce nanoparticles but contribute to larger particles. Common methodologies in studying these emissions include laboratory experiments (with brake dynamometers, tyre dynamometers, chassis dynamometers, and simulators), field tests (tunnel and real road emission tests), and source apportionments. The emission rate of PM_0.1_, calculated based on particle number concentration, ranges from 1.2% to 98.9%, depending on driving conditions. Extreme driving conditions result in high nanoparticle generation. Emission inventories reveal that PM_0.1_ emission levels have remained stable since 2020, without an observable reduction. Moreover, emissions attributable to brake wear are found to surpass those from tyre wear. Current mitigation strategies focus on material improvements for brake pads and tyres, better road maintenance, and regulatory measures. Mitigating the environmental and health impacts of nanoscale particulate matter requires additional research and regulations to control it at the source.

## Highlights

• Brake and tyre wear are important sources of non-exhaust nanoparticle emission.
• Nanoparticles are formed during brake and tyre wear at high temperatures.
• Measured nanoparticle concentrations vary significantly depending on driving conditions.
• Real-world measurements of non-exhaust nanoparticles are crucial for database creation.
• Regulations are needed to control non-exhaust nanoparticle emissions at the source.

## Graphical abstract

**Figure:**
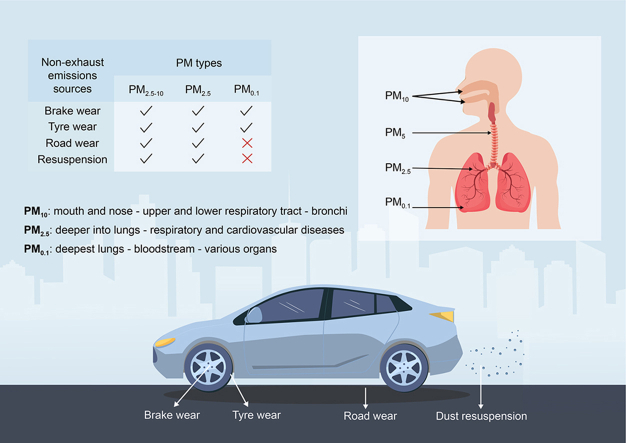

## 1 Introduction

The negative impact of vehicle emissions on air quality and human health is a significant concern worldwide. Rapid urbanisation and the widespread use of motor vehicles have exacerbated the deterioration of air quality [1]. These emissions are broadly classified into exhaust and non-exhaust emissions (NEEs) [2]. While exhaust emissions have received much attention in recent years due to their association with incomplete fuel combustion and lubricant volatilisation, the role of NEEs in exacerbating air pollution has not gained similar attention from researchers and policymakers [3]. The proportion of exhaust emissions has been reduced through the efficient development of exhaust emissions process control technology. As a consequence, NEEs constitute a significant proportion of total particulate matter (PM) emissions from driving, potentially accounting for up to 90% [4]. NEEs are a complicated combination of particles with varying sizes, compositions, and chemical characteristics, mainly comprising PM_10_. However, a considerable fraction of the emissions also contains PM_2.5_ [5]. Their smaller counterparts, also known as nanoparticles (<100 nm or PM_0.1_), are also generated but are often overlooked due to their nanoscale size, which makes them more difficult to measure compared with larger particles. Nanoparticles can cause significant harm to the human body as they can penetrate deep into the respiratory system and even cross biological barriers, causing inflammation and oxidative stress [6]. Nanoparticles are primarily produced through gas-to-particle conversion. During processes such as brake wear and tyre wear, heat and friction can lead to the production of particles [[7], [8], [9]].

The significance of these non-exhaust sources is increasingly acknowledged for their contribution to total PM emission [10]. The Euro 7 emissions standard is scheduled to be implemented in 2025 and is anticipated to be the final iteration of vehicle emissions regulations. This standard now considers NEEs, which have been relatively underregulated in previous policy versions [11]. The forthcoming standards are designed to significantly reduce vehicle emissions, including brake wear particles. This new standard aims to cut brake particle emissions from vehicles by 27%. These particles primarily fall under the categories of PM_10_ and PM_2.5_ due to the mechanical nature of brake wear, but restrictions on the total number of nanoparticles are also considered.

Table 1 summarises the past review papers about NEEs and extracts the findings about PM. These reviews have focused on NEEs from the perspective of understanding the sources, their impacts on humans and the environment, and the factors affecting NEEs, source identification, particle characterisation, and policy implications. Particularly, most studies analyse PM across all size ranges without specifically focusing on nanoparticles. Although nanoparticles are acknowledged within the scope of non-exhaust PM emissions and are noted to possess distinct physical and chemical properties, the emission characteristics of nanoparticles and their generation mechanisms require further analysis and investigation.

**Table 1:** Summary of review paper discussing various aspects of non-exhausted particles and the finding on particulate matter.

Study focus	Finding on PM	Ref.
This paper provides a comprehensive review of studies on nanoparticle emissions related to transportation sources, including both exhaust and non-exhaust emissions. It also discusses the impact of nanoparticle toxicity.	Significant variations in emission levels have been observed across different types of studies and measurement instruments used, and adopting advanced measurement methods reveals a substantial presence of nanoparticles. Direct abrasion typically generates coarse particles, whereas fine and nano-sized particles are formed in thermal processes.	[12]
This article provides an overview of the current state of research on non-tailpipe PM emissions and highlights areas where further research is needed to develop targeted policy responses to this issue.	Considering the exposure to NEEs and their impact on human health, current research is limited and necessitates further specific measurements of the sources of various NEEs. Present studies on emission measurements indicate that PM concentrations vary significantly due to a multitude of factors, resulting in a wide range of particle sizes being emitted.	[13]
This article analyses NEEs involves examining their various sources, formation mechanisms, and chemical and physical characteristics. Additionally, it presents effective mitigation strategies that are currently in place.	NEEs account for 90% of the total PM transport-related emissions; moreover, the contribution from resuspended dust exceeds that from brake wear, tyre wear, and road wear sources.	[4]
This article reviews numerical modelling methods for atmospheric NEEs, proposes reduction measures for engineering controls, road cleaning and re-suspension of dust suppressants, and describes future emission trends for NEEs	Particles are present in both fine and coarse fractions, with a predominant presence in the coarse fraction in terms of mass distribution. The inventories of PM emissions vary across different regions.	[14]
This article summarises current information on NEEs from road vehicles, describes effective techniques to minimise NEEs emissions, and proposes that international measuring methodologies should be developed to provide the basis for standards.	Quantitative data on NEEs are scarce, and the size distribution of particles across various dimensions exhibits a high degree of uncertainty.	[15]
This review compares the weight and PM emissions of internal combustion engine vehicles and electric vehicles.	The NEEs of vehicles are proportional to the vehicle's weight. Due to the impact of vehicle weight on PM emissions, the proliferation of electric vehicles cannot effectively reduce the total emissions of PM.	[5]
This review emphasises on the emissions factors of brake wear and tyre wear to discuss the mass size distribution, particle number distribution, chemical properties, and health aspects of NEEs.	The contribution of each source to NEEs is as follows: Brake wear accounts for 16%–55%, tyre wear for 5%–30%, and resuspension for 28%–59%. In terms of mass size distribution, the majority of particles generated from brake and tyre wear fall within the PM_10-2.5_ range. It was identified that there is PM_0.1_ emitted.	[16]
This review summarises major and recent discoveries from four complimentary research fields and seeks to identify gaps in present non-emissions research and policy.	In terms of mass distribution, PM pollution of resuspension is the primary contribution source, and its contribution to future PM concentrations is expected to continue increasing.	[10]

The overall goal of this paper is to systematically evaluate the existing research results on NEEs and extract relevant data and information related to nanoparticles. By reviewing the broader NEEs literature, this paper identifies experimental and analytical methods, as well as instruments that are applicable to nanoparticle studies, and clarifies future research directions in this area. The specific objectives are to: (i) summarise the sources of NEEs from road vehicles, focusing on factors that influence the formation and properties of nanoparticles; (ii) review the experimental methods used in NEEs research and summarise the emission rates of nanoparticles from these studies; (iii) analyse exposure risks associated with NEEs and propose strategies for mitigation; and (iv) identify research gaps in the study of nanoparticles within NEEs and suggest directions for future research that address nanoparticle emissions.

## 2 Scope and outline

**Fig. 1:**
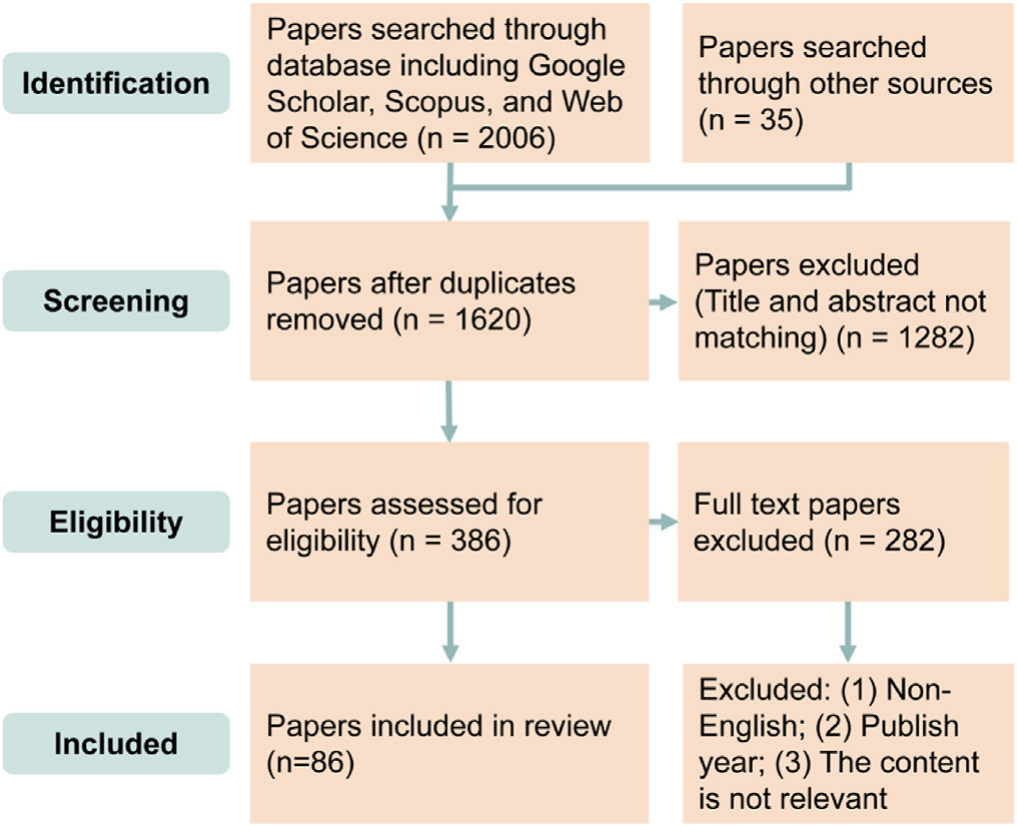
Literature screening process (2008–2023) using Google Scholar, Scopus, and Web of Science.

This article primarily investigates nanoparticles, particularly those found within NEEs from road vehicles. Larger-sized PM is not the primary focus of this study and is discussed only to provide context for various arguments. Various databases and search engines were used, including Google Scholar, Scopus, and Web of Science. A combination of relevant search keywords was included: “non-exhaust emissions”, “nanoparticles”, “PM_0.1_”, “brake wear”, “tyre wear”. The bibliographies of relevant articles were also analysed to identify other potential sources. The selected keywords align with the paper's research topic and define the scope of this study. The literature screening included articles published in English within the last 15 years. Fig. 1 shows the article screening process using the systematic review literature search method. Firstly, all relevant literature meeting the search criteria was collected from multiple databases, yielding a total of 2006 articles. The second screening step was performed to exclude the literature duplicated in the database and then further exclude again based on the title and abstract. In the third step, the content of the articles was read through to select the articles that fit this study. The fourth step finally identified 86 highly qualified articles for review. In addition, other relevant articles and reports that did not appear in the search but were found through cross-referencing were also discussed.

## 3 Sources, formation mechanisms, and characteristics

Recent research has investigated the sources of PM from NEEs, identifying brake wear, tyre wear, road wear, and resuspension as primary contributors [4,8]. Through an analysis of these main sources, direct sources of nanoparticle emissions have been identified, as depicted in Table 2. Analysis reveals that nanoparticles are predominantly generated from brake wear and tyre wear under conditions reaching critical temperatures, whereas they are not directly produced by road wear and resuspension. Recent experiments demonstrated that dry clutches emit nanoparticles during operation, with particles showing a bi-modal size distribution [17]. This study found that nanoparticles are generated during both the run-in and steady-state phases of clutch use. Further research using a novel test rig confirmed the emission of nanoparticles from dry clutches; in this study, two spectrometers were employed to analyse both the particle size distribution and chemical composition, again identifying nanoparticles with a similar elemental composition [18]. These findings suggest that dry clutches can emit nanoparticles; however, their overall contribution compared to primary sources like brake and tyre wear remains insufficiently studied and requires further investigation. This section analyses each emission source and elucidates the nanoparticle formation mechanisms and their characteristics. Brake wear and tyre wear can lead to nanoparticle generation. In contrast, road wear and resuspension primarily contribute to larger-sized particles that can settle or re-entrain into the air.

**Table 2:** Non-exhaust emission sources from the vehicles and the particle emission types for each source.

Non-exhaust emission sources	PM types		
	PM_2.5-10_	PM_2.5_	PM_0.1_
Brake wear	✔	✔	✔
Tyre wear	✔	✔	✔
Road wear	✔	✔	✖
Resuspension	✔	✔	✖

### 3.1 Sources and classification of particulate matter

PM originates from natural sources, such as dust and pollen, and anthropogenic sources, including automobile emissions, factories, and power plants [19]. PM can be classified into coarse, fine, and nano/ultrafine particles (0.1 μm; PM_0.1_).

Different particle sizes have varying degrees of impact, with nanoparticles generally posing greater health risks [20]. Due to their small size, nanoparticles can enter the lungs through the nose and throat and once inhaled, can penetrate deeper into the respiratory system, potentially leading to serious health consequences, including damage to the heart and lungs [21].

### 3.2 Measurement of nanoparticle number distribution

Understanding nanoparticle dynamics requires distinguishing between mass and number distribution due to their differential implications for health and environmental impact assessments. In the case of non-exhaust nanoparticles, the focus of analysis requires the number distribution rather than the mass distribution. Mass distribution, defined as the mass of particles per unit volume of air (μg/m³), is traditionally applied to assess the concentration of coarse and fine PM, but is insufficient for capturing the complexities of nanoparticles due to their minimal mass. This can lead to underestimations of their potential impacts [22]. Number distribution, indicating the total count of particles per unit volume, is a more crucial metric for comprehensively understanding nanoparticle behaviour [23]. It allows for the precise tracking and evaluation of the release, dispersion, and potential health and environmental impacts of airborne nanoparticles. For nanoparticles, number concentration is more pertinent than mass concentration because smaller particles, for the same mass, interact more effectively with biological surfaces [24]. Additionally, the transformation processes of nanoparticles include nucleation, coagulation, deposition, and condensation. Their number and size distributions change dynamically after release into the atmosphere [25]. The high mobility of nanoparticles and their tendency to form agglomerates require the use of number concentration to better characterise these particles [26].

Various instruments are commonly utilised to measure particle number distribution, each offering different levels of precision, which can result in variability in the measured data. Table 3, Table 4 summarise the instruments used in brake and tyre wear emissions studies, their measurement ranges and corresponding PM_0.1_ number concentration emission rates. The emission rates of PM_0.1_ show substantial variability across experiments, ranging from 1.2% to 98.9%, depending on the experimental setup, including measurement duration and sampling locations. For instance, during aggressive driving conditions such as full-stop braking, rapid acceleration, or extreme cornering, PM_0.1_ emissions were observed to increase exponentially, while during smooth and steady driving, these particles were undetectable [36]. Similarly, tyre wear emissions were investigated using a tyre test bench, and it was found that under conditions of longitudinal force and wheel load variations, the concentration of nanoparticles approximately doubled compared to that observed during tests involving variations in slip angle and speed [35]. These results show that extreme driving conditions significantly elevate nanoparticle emissions from road vehicles compared with significantly lower concentrations at steady cruising speeds, thus presenting a large disparity in emission rates.

**Table 3:** Different types of experiments of brake wear emission test.

Instrument	Measurement range	Experiment type	PM_0.1_ number concentration emission rate (%)	Ref.
ELPI, FMPS, OPC	0.005–10 μm	Chassis dynamometer	3.7	[27]
		On-board	32.8	
		Roadside	1.6	
ELPI	0.006–10 μm	Brake dynamometer	1.8–6.0	[28]
FMPS, OPS, ELPI	0.006–10 μm	Pin-on-disc	98.9	[29]
FMPS, OPS, ELPI	0.006–10 μm	Pin-on-disc	85.8	[30]
APS, SMPS	0.001–10 μm	Brake dynamometer	1.2	[31]

ELPI, electrical low pressure impactor; FMPS, fast mobility particle sizer; OPC, optical particle counter; SMPS, scanning mobility particle sizer, APS, aerodynamic particle sizer; OPS, optical particle sizer.

**Table 4:** Different types of experiments of tyre wear emission test.

Instrument	Measurementrange	Experiment type	PM_0.1_ numberconcentrationemission rate (%)	Ref.
FMPS, OPS	0.006–10 μm	Tyre wear simulator	34	[32]
APS, SMPS	0.001–10 μm	Road simulator	2.8–5.2	[33]
APS, SMPS	0.001–10 μm	Road simulator	24.7–73.2	[34]
EEPS	0.006–1 μm	Road simulator	77.8–83.3	[35]
EEPS	0.006–1 μm	On road test	40.4–98	[36]

EEPS, engine exhaust particle sizer.

To accurately assess the number concentration and size distribution of nanoparticles from non-exhaust PM, a variety of high-precision instruments have been used in recent studies. These instruments are essential for capturing particles across various size ranges. Instruments such as electrical low-pressure impactor (ELPI), fast mobility particle sizer (FMPS), optical particle counter (OPC), scanning mobility particle sizer (SMPS), aerodynamic particle sizer (APS), optical particle sizer (OPS), engine exhaust particle sizer (EEPS) are commonly used. Table 5 summarises the basic parameters of different instruments. Some experiments use multiple instruments in combination to extend the measurable size range of particles. Selection criteria for these instruments should align with the specifics of the experimental setup, including the intended indoor or outdoor environment, the study's duration, and the target particle size range. Instruments with insufficient lower-cutoff diameter are not able to measure the contributions of nanoparticles, leading to underestimations of their contribution and impact in PM studies.

**Table 5:** Comparison of instruments for particle number distribution measurement.

Instrument	Measurement range (nm)	Air flow (L/min)	Advantages	Disadvantages
ELPI	6–10,000	10	Wide size range, robust	Frequent maintenance, limited portability
FMPS	5.6–560	10	Quick measurements, field suitable	Lower precision compared to other instruments
OPC	300–10,000	0.05	Easy to use, cost-effective	Limited nanoparticle sensitivity
SMPS	1–1000	0.3	High resolution for size distribution	Limited to stable environments
APS	500–20,000	1	Handles large particles	Not suitable for nanoparticles
OPS	300–10,000	1	Cost-effective	Less accurate for smaller size ranges
EEPS	5.6–560	10	Real-time measurement, robust	Lower precision compared to the other instrument

### 3.3 Brake wear emissions

#### 3.3.1 Formation mechanisms

Brake wear emissions are generated during braking due to frictional contact between the brake pads and the rotating brake disc or drum. This process involves both mechanical wear and thermodynamic processes, contributing to the generation of PM across a range of sizes. Two primary mechanisms contribute to aerosol particle formation within the brake system [16]. First, mechanical wear of the sliding contact surfaces releases coarse and fine particles into the atmosphere [3]. Second, frictional heating of these surfaces leads to steam generation. The interaction between steam and air initiates the nucleation and subsequent aggregation of nanoparticles [37].

Two main brake system configurations are used: disc brakes and drum brakes. In disc brakes, flat pads press against a metal disc, whereas in drum brakes, curved shoes press against the inner surface of a rotating cylinder. The physical and chemical properties of emitted particles vary depending on the brake lining composition and the driver's braking style [13].

PM emissions from brake wear are influenced by braking parameters such as pressure, speed, and braking intensity. For example, urban driving, characterised by frequent braking, tends to produce more PM than highway driving. Notably, the quantity of nanoparticles increases during full stops compared to normal deceleration [16]. In addition, tests on a pin-on-disc tribometer have shown that temperature is an important factor in brake system emissions, with the nanoparticle fraction increasing as the system temperature rises [29]. The substantial heat generated during braking leads to the thermal and chemical degradation of brake linings, resulting in the formation of nanoparticles. It was found that PM_10_ accounted for 86% of the PM mass released by brake wear and 63% of PM_2.5_, while PM_0.1_ accounted for 33%. Nanoparticle production strongly correlated with the increasing temperature in cast iron discs [38]. Submicron particles are formed through evaporation/condensation processes and aggregation of primary nanoparticles rather than direct abrasive wear; the braking process, when the cast iron disc temperature exceeds 300 °C, leads to the generation of nanoparticles due to the thermal degradation of brake pad materials [30]. Nanoparticle generation becomes significant when temperatures reach 400 °C–500 °C [31].

In electric vehicles, regenerative braking has been proposed as a potential mitigation strategy to reduce brake wear and associated emissions. However, it was found that while regenerative braking reduces brake wear, the NEEs from electric vehicles remain comparable to those from internal combustion engine vehicles due to the increased vehicle weight [5]. The emission factors (EF) of different types of vehicles were evaluated, revealing that the NEEs of electric vehicles might even exceed those of conventional vehicles depending on factors such as the extent of regenerative braking, road type, and vehicle model [39]. It has been pointed out that although regenerative braking decreases PM emissions by reducing brake wear, the additional weight of electric vehicles may offset these reductions [40]. Thus, the overall NEEs must be evaluated in the context of multiple contributing factors.

Recent studies have reported significant differences in PM emissions from brake friction between electric vehicles and conventional fuel vehicles. Nanoparticle emissions were found to increase when brake pad temperatures exceeded 170 °C in gasoline vehicles, while regenerative braking can maintain lower temperatures, reducing nanoparticle emissions [41]. This threshold was identified through direct measurements of brake pad surface temperatures using thermocouples. It was observed that nanoparticle emissions from three different brake materials, specifically low-metallic, non-asbestos organic, and cast iron, varied with the surface temperature of the brake pads and discs [42]. For example, in the tests with a low-metallic brake pad, no nanoparticle emissions were detected when the pad surface temperature was at 150 °C. However, when the pad surface temperature exceeded 160 °C, nanoparticle concentrations in the 1.3–4.4 nm range rose to approximately 60,000 particles/cm³.

#### 3.3.2 Chemical characteristics of brake wear particles

Brake linings can be made of several different material combinations. They generally comprise five main components: fibres, abrasives, lubricants, fillers, and binders [9]. These materials significantly influence the chemical characteristics of particles generated during braking. Studies have shown that brake wear particles contain a complex mix of metals, organic compounds, and inorganic materials [3]. Understanding the composition of both brake wear debris and the brake lining materials is crucial to comprehending how the chemical nature of PM changes during the braking process.

In the past, brake pads contained asbestos, but due to its carcinogenic properties, modern brake linings now use composite materials. They mainly consist of metals and metal-containing inorganic compounds (e.g., sulphides, oxides, silicates), including non-asbestos organic (NAO), semi-metallic (SM) and low metal (LM), ceramics (used on some expensive vehicles). These materials mainly consist of metal compounds such as iron (Fe), copper (Cu), zinc (Zn), and historically, lead (Pb), although lead is no longer used due to health risks [38]. Fe typically constitutes 30%–60% of the total metal content in brake wear particles, depending on the brake lining material. Cu accounts for approximately 1%–10%, while Zn content varies and is usually less than 5% [9]. Manganese (Mn) and silicon (Si) are present in smaller proportions, each contributing less than 1% to the total particle composition [16]. The concentration of these metals can vary significantly depending on the manufacturer and the type of brake lining used.

High temperatures during braking cause chemical changes in the brake lining materials, resulting in the formation of metal oxides, such as Fe₂O₃ and CuO, which are more common in nanoscale particles [31]. Organic carbon (OC) and elemental carbon (EC) are also present in brake wear particles, the presence of these carbonaceous components is influenced by the organic materials used in brake pads and the temperatures generated during braking [43].

Braking occurs under high temperature and pressure conditions, causing alterations to the chemical properties of the lining material [16]. However, certain heavy metal components within brake linings maintain stable forms distinct from those in other sources. Based on this, some studies utilise certain elements as the brake wear tracers. Elements such as Fe, Ba, and Cu are commonly used as tracers to identify brake wear particles in environmental studies. The most common tracers for brake wear particles and the instruments used to measure them are shown in Table 6. These tracers are frequently detected using techniques such as X-Ray Fluorescence (XRF), inductively coupled plasma mass spectrometry (ICP-MS), and atomic emission spectrometry (ICP-AES).

**Table 6:** Commonly identified trace elements and compounds in PM emissions resulting from automotive brake and tyre wear.

Brake wear	Tyre wear	Measurement methodology	Ref.
Fe, Ba, Cu, Sn	Zn	ICP-OES, ICP-MS, XRF	[44]
Fe, Zn, Mn, Cu, Ni		ICP-MS, XRF	[45]
Fe, Ba, Zr, Cu, Ti	Si, Zn	ICP-MS	[46]
Fe, Cu, Zn, Cr, Sn, Sb	Zn	ICP-MS, ICP-AES	[47]
Ti, Cu, Ba, Fe	Zn, Cd	ICP-AES, ICP-MS	[48]
Zr, Cu, Ba, ti, Cr, Fe	Zn, Al	XRF	[49]
Fe, Cu, Ba, and Sb		ICP-MS	[50]
Ba, Cu, Fe, Sb	Zn	ICP-MS	[51]
Fe, Cu, Sn, Zn		ICP-AES, XRSF	[31]
Sb, Cu, Fe, Pb	Zn, Co	ICP-MS	[52]

ICP-OES, inductively coupled plasma optical emission spectroscopy; ICP-MS, inductively coupled plasma mass spectrometry; XRF, X-Ray Fluorescence; ICP-AES, inductively coupled plasma atomic emission spectroscopy.

### 3.4 Tyre wear emissions

#### 3.4.1 Formation mechanisms

Tyre wear particles are generated through various mechanisms associated with tyre-road interactions, including the shear and friction forces that occur during steering, braking, and driving actions [53,54]. These interactions are influenced by mechanical, thermomechanical, and thermochemical processes. Mechanical actions result in the emission of coarse particles, whereas thermal degradation, volatilisation, and condensation at elevated temperatures lead to the production of fine particles [55]. These fine particles are subject to further transformation through aerosolisation and condensation of tyre material, significantly contributing to non-exhaust nanoparticle emissions in the ambient air through gas-to-particle conversion processes [9,54].

Laboratory experiments further indicate that organic tyre material evaporates and subsequently re-condenses onto small particles [56,57]. A study found that the use of winter tyres in colder climates results in increased tyre wear and particle emissions [56]. The aggressive tread patterns and softer rubber compounds of winter tyres enhance the interaction with harder road surfaces common in winter conditions. This interaction contributes to increased mechanical and thermal processes that lead to the formation of nanoparticles. Additionally, the roughness of the road surface acts as a critical effect factor, influencing the rate and characteristics of tyre wear. Research indicates that approximately 80% of PM generated from tyre wear is deposited on the road surface or adheres to the tyre rubber due to surface interactions and static electricity, with the remaining 20% becoming airborne and contributing to non-exhaust PM pollution [58].

#### 3.4.2 Chemical characteristics of tyre wear particles

Tyres are made from a variety of materials, including synthetic rubber, fillers, plasticisers and other chemicals [58]. Particles generated from tyre wear are a complex mixture of rubber debris and other particles originating from various sources [4]. The main components of tyre treads are carbon black, elastomeric compounds, steel wires, fibres, and other organic and inorganic compounds [4]. The chemical elements and characteristics of tyre wear PM vary due to heat, wear, and road material. Heat and shear forces between the tread and the road surface mainly determine the wear process [3,59,60].

OC and EC are significant constituents of tyre wear particles. OC accounts for approximately 10% of the PM_10_ mass, originating from the breakdown of rubber polymers and additives, while EC, primarily from carbon black, comprises about 1.5%–3.3% of the PM_10_ mass [33]. The zinc content of tyre manufacturing materials constitutes approximately 1%, while the zinc content of PM generated by tyre wear is expected to account for 23%. Hence, tyre wear is an important source of zinc in the environment [60]. Based on the elements used for tyre wear tracer in several studies as shown in Table 6, Zn is predominantly utilised as the tracer element [44,48]. Approximately 70% of the emitted nanoparticles were found to be semi-volatile, likely originating from vapourisation events during tyre-road interactions [61]. These findings suggest that nanoparticles generated from tyre wear not only contain common elements such as Zn but also exhibit complex chemical transformations due to high temperatures and mechanical forces.

### 3.5 Road wear emissions

#### 3.5.1 Formation mechanisms

Road wear PM is generated by friction between the road's concrete particles and the tyre's surface, releasing a wide range of particle sizes [3]. The exact size distribution of particles emitted from road wear can vary depending on various factors, such as the type of road surface, tyre materials, and driving conditions. As other sources of pollution can have similar or identical compositions, they can mix with the PM produced by road wear and mix within the tyre rubber [9]. Furthermore, the friction between the tyre surface and the road surface causes the tyre to wear down. Also, it tends to wear down the road surface, especially where the road surface has broken up. As a result, PM formed by road friction is released into the atmosphere. The complex mix of components makes it more challenging to identify PM from road wear, and field measurements cannot clearly distinguish road wear particles from road resuspended particles. Determining a proper tracer for road wear has proven difficult [58]. Previous report indicates that the mass size distribution of road wear particles peaks around 6–7 μm, with no particles observed below 0.5 μm [62]. This indicates that physical wear predominantly generates fine and coarse particles during road wear without directly producing nanoparticles.

#### 3.5.2 Chemical characteristics of road wear particles

Two types of street pavement are used in urban areas: bitumen and concrete. Bitumen mainly comprises 95% mineral materials; the rest is primarily filler and bituminous binder. The mineral particles produced by road materials mostly contain iron, aluminium, calcium, silicon, and potassium. To analyse the complex composition of these particles, controlled laboratory experiments can be used to simulate real-world conditions and provide a clearer understanding of the chemical makeup. The particle size distribution measured under controlled laboratory conditions may closely reflect the distribution of road wear particles found in the environment [9].

### 3.6 Resuspension emissions

#### 3.6.1 Formation mechanisms

Another source of PM entering the air from NEEs is the resuspension of road dust. Resuspended PM is formed when dust from various sources of pollution accumulates on road surfaces. There are several possible sources of resuspended particles: dry and wet deposition of particles in the air; particles deposited by vehicle wear; particles that already exist on the road surface are suspended into the air during driving; tyre grind dust, resulting in coarse particles turn to be smaller particles [51]. At the same time, the effect of wind will also cause the resuspension of surface dust. Resuspended PM includes the sources mentioned before: brake wear, tyre wear, road wear and several other sources that produce PM deposited on the road, in addition to the more complex components. Measurements of resuspension emissions show that these particles mainly fall within the PM_2.5-10_. For example, resuspended particle emissions were measured, and their mass size distribution was found to range from 0.4 μm to several tens of micrometers [63]. The mass modal diameter of resuspended particles was reported to be approximately 5 μm [51].

#### 3.6.2 Chemical characteristics of resuspension particles

The composition of road dust is linked to crustal material, with the specific composition being related to geological conditions, producing significant variations in different locations, and being influenced by seasonal changes [9,26]. Road dust often contains significant metals such as copper, zinc and cadmium. The presence of pollutants in road dust and roadside soils provides a method by which particles generated by anthropogenic sources can be separated from natural crustal sources by calculating the crustal enrichment factor. However, other methods need to be developed to distinguish the relative contribution of road wear and heavy overhanging road dust. The complex composition of resuspended PM makes finding a representative tracer species unlikely.

## 4 Experiments of NEEs

The measurement, quantifying the amount of NEEs produced and understanding the distribution of NEEs can help further investigate their origins and the contribution of NEEs to the PM found in contaminated ambient air. However, because there are different PM sources in the environment, it is challenging to quantify and identify them [51]. Currently, NEEs are largely examined through controlled laboratory research and outdoor field experiments. Furthermore, the degree of road, tyre and brake wear may be examined in a laboratory. Brake wear may be tested using a brake dynamometer under carefully controlled conditions [13]. This section summarises the laboratory and field study methods, the emission factor measurement and emission inventory, and the source apportionment.

### 4.1 Laboratory experiments

Laboratory experiments have the advantage of being flexible in operation, making it possible to set specific conditions and able to exclude certain environmental disturbances. Studies have used varied simulation methods: testing the driving characteristics of cars on real roads, simulating the operation of brake pads and tyres, and carrying out tests on different road simulators. With the introduction of new vehicle emission standards, such as Euro 7, laboratory tests are increasingly adopting the UNECE (United Nations Economic Commission for Europe) measurement protocols. These standards aim to ensure consistency and comparability across experiments, especially regarding brake and tyre wear particle emissions.

The primary experiments conducted using rigs in the laboratory studies include the brake dynamometer, the chassis dynamometer, the tyre dynamometer, and the road simulator. For instance, experiments were conducted using a brake dynamometer that simulated a vehicle's moment of inertia with a rotating weight. The entire brake system was enclosed in a chamber to eliminate ambient PM interference, with particle-free clean air supplied through a high-efficiency PM air filter [64]. The brake wear PM emission factor per vehicle was calculated based on a specific brake force distribution. Additionally, a road simulator was used to study the wear particles generated by the interaction between two tyre brands and a composite pavement surface [33]. Particle size distributions were monitored using Scanning Mobility Particle Sizer, with continuous measurements of particle mass concentrations. Despite laboratory tests providing precise control over material composition, driving cycles, and specific variables, factors such as the variation in brake and tyre materials, driving speeds, and patterns remain challenging to replicate under laboratory conditions. Furthermore, chassis dynamometers are used to simulate real driving conditions to measure brake and tyre wear emissions under different driving conditions. For example, a chassis dynamometer was employed to assess non-exhaust particle emissions from brake and tyre wear. It was observed that emissions of brake wear particles and tyre-road contact particles significantly increased during periods of acceleration and deceleration compared to constant-speed driving; managing driving speeds to not exceed 70 km/h and ensuring smoother traffic flow could effectively mitigate NEEs [27].

Component analysis of NEEs provides information on their chemical composition, facilitating the differentiation between various types of emissions [4]. However, the physical and chemical properties of NEEs, such as their difficult handling, heat sensitivity, and solubility in water, pose challenges for traditional PM analysis methods like ion chromatography, gas chromatography, and aerosol mass spectrometry [65]. Especially for carbonaceous components, selective analysis is challenging due to their non-volatile or insoluble nature. For metal components, some studies have resorted to X-ray spectroscopy for analysis [13].

The detection of tyre-road wear particles is challenging. There are two main groups of methods: single-particle and bulk studies. Typical methods for single particle measurement use features such as shape, surface roughness, tactility, colour, and chemical markers associated with inorganic or organic components [58]. Common single-particle methods include light microscopy, Scanning Electron Microscopy with Energy Dispersive X-ray Analysis (SEM-EDX), and Raman Spectroscopy. Additionally, various infrared light measurement techniques such as Micro Fourier Transform Infrared (μFTIR), Attenuated Total Reflectance Fourier Transform Infrared (ATR-FTIR), Simultaneous Thermal Analysis Fourier Transform Infrared (STA-FTIR), and Thermogravimetric Analysis Fourier Transform Infrared (TGA-FTIR) spectroscopy have gained popularity for their ability to provide detailed chemical characterisation at the single-particle level [65]. Bulk methods typically involve analysing the mass of particles collected onto the filters to determine average properties and overall chemical composition. Common bulk analysis techniques include Inductively Coupled Plasma Mass Spectrometry (ICP-MS), gravimetric analysis, X-ray fluorescence (XRF) spectroscopy; these bulk analysis methods provide valuable data on the concentration and distribution of particles [66]. In some cases, combining both bulk and single-particle methods allows for a more comprehensive understanding of PM characteristics.

### 4.2 Field tests

#### 4.2.1 Tunnel test

Tunnel testing is a useful method in NEE studies, measuring the emissions of various indicators as the vehicle drives through a tunnel. The tunnel test approach provides a comprehensive measurement that includes different sources and allows more factors to be taken into account when measuring under realistic conditions. Tunnel measurements are also reproducible, easy to perform, and can be used to compare different types of emissions from different vehicles. An in-depth study of non-exhaust PM emissions from vehicles in major Chinese cities was conducted using tunnel experiments [46]. Actual EF for various components of NEEs were measured, showing that EF vary from city to city and vehicle type to vehicle type, suggesting the need for city and vehicle-specific strategies to reduce NEEs. Measurements were conducted at a city centre bus station, representing an open environment, and in a motorway tunnel, representing a semi-enclosed environment, using SMPS to study the contribution of nanoparticles [67]. The results indicated that at the bus station, the number concentration of 50–100 nm particles peaked at approximately (1.5–2.0) × 10^4^ particles/cm^3^, while the number concentration of 20–50 nm particles inside the tunnel was about 2.0 × 10^4^ particles/cm^3^, predominantly within this size range. This research suggested that the concentration of nanoparticles in the open environment is slightly higher than in the semi-enclosed environment. In the open area measurement, the inflow of external air can cause particles and pollutants from vehicles to combine; within the tunnel, vehicle traffic is the primary source of nanoparticles, providing a controlled setting that is beneficial for emission measurements and elemental analysis.

#### 4.2.2 Real road emission test

Laboratory experiments can simulate road conditions under isolated conditions; while laboratory tests allow individual variables to be examined under controlled conditions, they may not fully account for the interactions between different variables that occur under actual road conditions. For example, in the case of resuspended dust, laboratory simulations must consider additional environmental factors such as wind speed, traffic volume, humidity and ambient temperature. PM from actual vehicles on the road involves both tyre rubber and road materials, so only considering the composition of tyre rubber may underestimate the effect of tyre wear on the concentration of airborne PM [13]. Therefore, some experiments were carried out on actual roads to improve the accuracy and comprehensiveness of the results. Sigma-2 passive samplers combined with single particle SEM/EDX were used to quantify and characterise airborne tyre wear particles along roadsides with different traffic volumes and speeds. This study revealed that the mass fraction of tyre wear particles at urban sites near busy roads was significantly higher than at urban background sites [68].

In addition, using the roadside air detection method is typical of road tests for NEE studies. Comparing roadside concentrations in a particular city with similar background measurements makes it possible to obtain size-resolved data as well as mass concentrations of PM components [14]. For example, the contributions of NEEs were estimated by collecting data from roadside sampling. Size-fractionated samples of airborne PM were analysed for size distribution and tracer elements. It was found that brake wear, tyre wear, and resuspended sources contributed 55.3%, 38.1%, and 10.7%, respectively, to the particle mass in the range of 0.9–11.5 μm [51].

Some of the approaches entail positioning air monitoring equipment near highways and collecting air samples for examination [69,70]. The air samples are tested for PM, nitrogen oxides, and carbon monoxide. The benefit of roadside air detection is that it can monitor pollutants from a more significant geographic region rather than being restricted to a single tunnel or stretch of road. This approach can also give information on the temporal and geographical distribution of NEEs. Nevertheless, there are certain limits to roadside air monitoring, such as being impacted by ambient factors like wind speed and direction and potentially catching emissions from non-vehicular sources.

### 4.3 Emissions factors and emission inventory

Researchers and regulators use EF to estimate the emissions of specific pollutants from individual vehicles or fleets. Functional relationships can be used to forecast the driving distance of vehicles or fleets and the number of pollutants emitted when a certain amount of energy is consumed, or a certain amount of fuel is used. EF are usually aimed at the type of vehicle and depend on vehicle characteristics, vehicle emission control technology, the type and quality of fuel, operating environment conditions, and other parameters [46]. Emissions vary considerably depending on brakes, tyres, road materials, and driving style. As a result, there is a wide range of uncertainty in the EF present in the NEEs, including broad variability between PM_10_, PM_2.5_ and PM_0.1_ size fractions.

To ascertain the EF from non-exhaust sources and their contribution to atmospheric particle concentrations, two primary approaches can be used: direct measurements and receptor modelling. Direct measurements can be conducted under real-world conditions or within a laboratory setting. Although this method offers a limited number of EF data points for vehicles, it benefits from being conducted under well-controlled conditions. However, replicating realistic braking scenarios for worn brakes in a controlled testing environment presents challenges. Conversely, receptor modelling requires accurate knowledge of the source composition and assumes that the specified source is relevant for the species measured at the receptor [71].

As brake wear is not easily performed under simulated conditions, the influence factor for brake pad wear is derived by direct measurement but has limited application depending on the type of vehicle, and it requires a complete understanding of the components to create the modelling. EF from tyre wear can be measured and modelled directly. A study indicated that tyres could lose 10%–20% of their mass during the lifetime [3].

Emissions inventories are emission estimates for different countries in specific application areas, including estimates of PM emissions from tyre wear, brake wear and road wear, and in some cases, also think about the road dust in suspension. Most countries follow the methodology for estimating emissions of the EMEP/EEA Guidance Manual for Air Pollutant Emission Inventories [72].

The California Air Resources Board (CARB) has conducted extensive studies on brake and tyre wear emissions, which outlines emissions under controlled and real-world driving conditions, providing emission rates and influencing factors information, such as brake wear PM emission rates can range from 3.3 to 13.6 mg/mile [73]. Braking EF for light-duty vehicles were summarised by comparing average values from various studies with roadside measurements and emission inventories to estimate the contribution of brake emissions to air pollution [25].

**Fig. 2:**
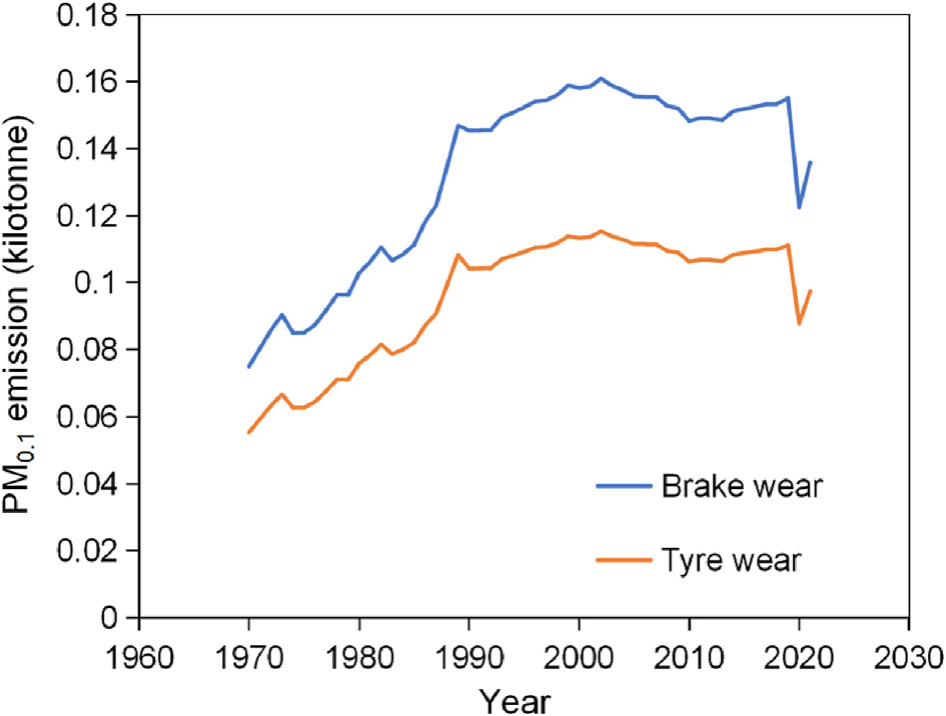
PM_0.1_ emission from brake wear and tyre wear of urban driving from 1970 to 2021 [74].

Emission inventory of UK has calculated PM_0.1_ emissions. It distinguishes between different types of vehicles, including passenger cars, light-duty, and heavy-duty vehicles, as well as considering various road conditions: motorways, rural roads, urban driving. For instance, PM_0.1_ data derived from brake and tyre wear on urban roads between 1970 and 2021, as recorded in the National Atmospheric Emissions Inventory in Fig. 2 [74]. This data indicates a significant upward trend in PM_0.1_ emissions between 1970 and 1989. From 1990 to 2019, the emission levels remained stable, and a notable decrease was observed in 2020. The significant decrease in 2020 is due to direct reductions in emissions because of reduced vehicle use during the COVID-19 epidemic. However, over the past few decades, nanoparticle emissions have been effectively controlled primarily due to the optimisation of brake pad and tyre materials. The transition from asbestos brake pads to low-metallic and non-metallic materials produces fewer particles due to lower wear rates [16]. Additionally, advancements in automotive manufacturing have focused on reducing vehicle weight, thereby decreasing the force required for braking and subsequently reducing brake wear [75]. In addition, relevant regulations have also contributed to controlling PM emissions, such as the Euro 7 standards discussed in Section 1. Despite the lack of growth, there has not been a significant reduction in PM_0.1_ emissions. In addition, PM_0.1_ emissions from brake wear are generally higher than from tyre wear.

The emissions inventory provides a clear depiction of emission trends but also simplifies the complex influences of vehicle size, type, technology, driving styles, and road conditions. Therefore, they are only available for the particular areas. On the other hand, emission inventories for non-exhaust sources are subject to considerable uncertainty because of the difficulty of measuring their emissions and distinguishing their sources. The emission inventory must approximate and average their effects or parameterise these factors in a relatively simple way. In the process of developing an NEE emissions inventory, the full range of emission sources and the factors that influence them need to be considered [76].

### 4.4 Source apportionment

Identifying and quantifying the contributions of different emission sources to airborne emissions is challenging due to the complex composition of these emissions. One effective technique for determining the origins of pollution is source apportionment. Several studies have utilised this method to analyse PM sources by considering the various pollution sources and their PM concentrations in specific areas and at specific times [77]. Common methodologies for PM source apportionment include Positive Matrix Factorization (PMF), Chemical Mass Balance (CMB), and Principal Component Analysis (PCA).

PMF is particularly useful in situations where data is limited and sources are complex, as it can effectively extract preliminary information about pollution source distribution. PMF was conducted on PM_0.1_ mass concentration data in California, where it was found that brake wear accounted for 1.7% of the PM source [78]. In another study, PMF was applied to PM_0.1-10_ mass concentration data separately for winter and summer in Japan [79]. The results revealed that during winter, no non-exhaust sources were identified. However, during summer, 4.1% of the PM_0.1_ came from tyre wear, while 0.6% originated from brake wear. Existing studies predominantly utilise mass concentration data of PM. The consideration of number concentration remains an area requiring further investigation. Furthermore, the sources' distribution rates are varied across different locations and times. PCA is a versatile statistical method that simplifies large datasets into more manageable subsets. PCA was applied on PM_10_ data to identify that NEEs account for 49% [80].

Comparing these three approaches of source apportionment, CMB requires detailed information on the chemical composition of each pollution source to match environmental pollutant concentrations with known sources. PCA is advantageous for identifying the primary patterns and trends in pollutant emissions. However, in terms of nanoparticle emissions, where the sources in the atmosphere are complex and uncertain, PMF is capable of distinguishing between various sources and providing information on their respective contributions.

## 5 Exposure risks associated with NEEs

NEEs, particularly nanoparticles, are associated with numerous health risks [13]. Particle size is an essential factor influencing particle deposition in the respiratory tract [3]. The physicochemical properties of NEEs indicate that if PM is inhaled through activation of airway inflammatory cells or interaction with underlying epithelial cells, it can potentially cause adverse biological responses. Coarse particles are concentrated in the upper respiratory tract, while fine particles penetrate deep into the abdomen, potentially affecting breathing, cardiovascular disease, respiratory disease, and lung cancer [4]. Studies have shown that nanoparticles from air pollution can enter the bloodstream through the respiratory system and potentially reach sensitive organs such as the brain, liver, and kidneys, where they may cause oxidative stress and inflammation. There is emerging evidence linking air pollution nanoparticles to neurodegenerative diseases, such as Alzheimer's, due to their ability to cross biological barriers [[81], [82], [83]]. Nanoparticles have a larger surface area per mass than their larger counterparts having the same chemical composition, which may make them more biologically active and potentially more harmful [84]. This increased surface area allows for greater interaction with biological tissues, potentially leading to oxidative stress, inflammation, and other toxicological effects [6,18].

In addition to general particle toxicity, specific components of NEEs, such as metals, can pose significant health risks. Higher concentrations of zinc in tyre PM leachate led to increased toxic reactions, thus indicating that zinc is one of the metals observed to have toxic effects [3]. Zinc is generally associated with adverse human health effects, particularly acute respiratory reactions. One study identified the impact of PM metals on emergency room admissions for asthma and respiratory problems and observed a significant association between emergency room admissions for asthma and zinc in PM [85]. This finding underlines the potential health risks associated with metal-rich NEEs, particularly as traffic-induced NEEs become a more prominent source of urban PM.

Beyond zinc, other metals such as copper, iron, and nickel, commonly found in brake and tyre wear particles, are associated with various health issues. Copper and iron contribute to the generation of reactive oxygen species, which results in oxidative stress and inflammation in respiratory tissues [86]. Nickel exposure is known to cause allergic reactions and respiratory ailments [87]. The toxicity of these metals may be amplified when they exist as nanoparticles due to their greater surface area and reactivity.

## 6 Mitigation measures

Various parameters influence NEEs, and the measures to reduce emissions can be derived from influencing parameters. Compared to other PM, nanoparticles are more difficult to control by conventional methods, so strategies to reduce nanoparticles are more focused on preventing their generation. This section summarises some of the potential measures that can reduce NEEs, describing technologies that are of practical importance.

### 6.1 Brake wear

The development of brake system technology has been driven by commercial interests, including friction characteristics, noise and vibration characteristics, wear, and durability. In the past, manufacturing processes were primarily concerned with their performance, while emissions were rarely put into design considerations. Little research has thoroughly focused on opportunities to reduce PM emissions through brake system design, material formulation and additional technologies [62]. Some car manufacturers are currently working on developing braking systems with good wear characteristics and good properties regarding the dirt on nearby surfaces. In conjunction with some of the available literature, several technologies and methods exist that can reduce PM from vehicle braking sources by (i) reducing particle generation; (ii) capturing particles after formation; and (iii) altering the particle properties after formation (e.g., changing the size distribution through enhanced agglomeration) can reduce the negative effects of particles [10]. There are new explicit limits on certain heavy metals in some locations, including copper, chromium and cadmium, and asbestos in brake pads is banned in most parts of the world, thereby reducing heavy metal emissions from brake wear. Technology is also being developed to capture brake wear particles at the source through filters mounted on brake discs [13]. Brake materials are expected to change significantly in the future, and tracking composition and tracers are essential for distribution studies and identification of environmental samples. Specifically for nanoparticles, exploring alternative materials, such as advanced organic composites, presents potential advantages in reducing wear rates and nanoparticle generation. Promoting smoother braking and less aggressive driving can help mitigate nanoparticle formation. Increasing public awareness of nanoparticles and encouraging environmentally friendly driving behaviours are also viable measures for reducing nanoparticle emissions. During braking, when the brake pads reach a certain temperature, the brake discs' thermal behaviour impacts PM emissions, which increase as thermal conductivity decreases [88]. Thus, managing the thermal behaviour of braking systems emerges as an effective strategy for reducing PM emissions. Moreover, friction material formulations also have an impact to some extent. Adopting new friction material formulations and selecting relatively safe components can reduce toxic and harmful material additions. This can prevent their emissions from entering the air [62].

### 6.2 Tyre wear

The use of hard rubber tyres can reduce wear but also increase noise, so low-wear tyre materials and constructions are used based on a balance between safe friction levels and the performance of environmental factors such as noise and rolling resistance. The selection of superior tyre materials can further mitigate the emission of particles [13]. Rubber constitutes a major contributor to microplastic pollution, and it is worth noting that nanoplastic pollution may also be a concern due to the further breakdown of microplastics. Therefore, ongoing research is committed to discovering alternatives to synthetic and natural rubber that are less likely to degrade into nanoplastic particles [59]. In addition to material innovations, tyre design improvements play a crucial role in minimising emissions. Low-wear tyre designs that optimise tread patterns and structural integrity can reduce the rate of wear while maintaining safety and performance. Such designs aim to balance friction, durability, and environmental impact, thereby contributing to the reduction of nanoparticle emissions. In terms of driver behaviour, measures such as minimising acceleration, maintaining appropriate tyre inflation pressure, and ensuring proper wheel alignment can reduce tyre wear, thus generating nanoparticle emissions [2].

### 6.3 Road wear and resuspension

There are some existing measures that can be implemented on roads to reduce PM emissions. These measures primarily target coarse particles generated from road wear. For instance, reducing the use of studded tyres and adopting smaller wheel circumference tyres can reduce road wear [13].

In addition to road wear, road surface conditions also affect PM emissions as road surface conditions can affect the extent of PM resuspension. Road maintenance measures have been implemented for a considerable period to reduce debris and loose material on the streets. Road cleaning usually involves the removal of vegetation, dirt, and litter to maintain aesthetics, hygiene, and the maintenance of drainage systems [89]. Technologies to achieve this include sweeping systems, vacuum systems, and street cleaning [90]. The use of mechanical sweepers has become a modern trend. Sweepers have been found to be effective at removing larger visible particles from street surfaces; however, there is evidence of a threshold effect, as sweepers cannot remove low dust loads [91]. More optimal results were obtained by performing water washing and sweeping together.

### 6.4 Vehicles manufacturing and legislation

During the vehicle's manufacturing process, the choice of materials can also impact the emissions during its application. For example, reducing the vehicle's mass, especially for electric vehicles through changing dimensions, design, materials, and battery technology, can reduce NEEs [62]. The choice of lighter-weight materials has some environmental implications and material recyclability issues, so specific assessments are required to select the most appropriate material.

The Euro 7 regulations, set to be enforced in 2025, introduce new provisions to measure PM emissions from brake wear and tyre wear. Some existing measures, for instance, the European Commission has mandated the development of a method to measure tyre wear rates as part of the tyre labelling regulations [92]. While this indirectly aids in managing emissions from tyre wear, the establishment of comprehensive regulations in the future could help standardise these emissions and further mitigate the environmental impact of NEEs.

## 7 Summary, conclusions, and future outlook

This review provides an overview of the sources, formation mechanisms, characteristics, measurement methods, experimental methods of NEEs from vehicles and extracted information about nanoparticles. It discussed the exposure risks associated with the NEEs and proposed the potential mitigation measure, thus highlighting the grey areas for further research. The following conclusions are drawn:

1) Through the analysis of the different emission sources of NEEs, it has been found that the primary sources generating nanoparticles include brake wear and tyre wear. In these processes, nanoparticles are produced through the gas-to-particle conversion mechanism occurring under high-temperature conditions resulting from varying driving conditions and environmental backgrounds. In particular, the formation of nanoparticles increases significantly once the temperature is over the critical temperature.

2) The emission rates of PM_0.1_ from brake wear and tyre wear range from 1.2% to 98.9%, largely influenced by driving conditions. Under extreme driving scenarios, nanoparticles are generated in large quantities compared with when the vehicles operate under steady cruising speeds when the production is significantly lower.

3) Common methodologies in studying NEEs include laboratory experiments (brake dynamometers, tyre dynamometers, chassis dynamometers, simulators, and chemical composition analysis), field tests (tunnel tests, real road emission tests), and source appointments (CMP, PMF, and PCA). More nanoparticle number distribution emission data is still needed under different driving conditions to understand the mechanisms of nanoparticle formation.

4) Measuring nanoparticles requires the use of sophisticated instruments due to their nanoscale dimensions, including ELPI, FMPS, SMPS, and EEPS, which can capture the nanoscale particles.

5) Due to the characteristics of nanoparticles, their post-generation treatment poses significant challenges. Therefore, the measures to reduce them mainly focus on decreasing their production from the source. This primarily includes improving the materials of brakes and tyres, refining driving styles, optimizing vehicle materials and designs, as well as implementing legislative measures.

Addressing the challenge of NEEs, particularly airborne nanoparticles from road vehicles, necessitates a multifaceted approach. Based on the conclusions above, the following recommendations are proposed:

**Further studies on non-exhaust nanoparticles are needed**. More relevant data needs to be collected for a more profound understanding of nanoparticle properties and emission information. This includes, for instance, the EF of nanoparticles under various emission scenarios and the inclusion of nanoparticle emissions in emission inventories. While substantial research has been conducted on non-exhaust PM, there remains a notable gap in the focused study of nanoparticles. Future research should therefore focus more on understanding nanoparticles in NEEs to reduce the overall risk and environmental impact of NEEs.

**Explore the formation mechanisms of nanoparticles under various conditions**. Current research indicates that nanoparticles are generated under high temperatures and extreme driving conditions. However, the specific effects of wear conditions, brake pad and tyre materials, and the impact of these factors during driving on emissions still require further investigation.

**Measure NEE nanoparticle distribution and analyse the source apportionment in the environment**. Detailed investigations into the composition, sources, and contributions of these nanoparticles to air pollution are needed to further comprehend their influence. More precise measurement and modelling solutions are required to quantify accurately their contribution to deteriorating ambient air quality.

**Future experimental designs should focus on developing standardised methods for nanoparticle measurement to ensure consistency and comparability across studies.** Incorporating real-time monitoring technologies can enhance the detection and analysis of transient nanoparticle emissions.

**Fostering innovation in vehicle design and materials**. Non-exhaust nanoparticles originate from brake and tyre wear, and improvements to brake and tyre materials can effectively reduce their production. In addition, the rational design of vehicles using lightweight materials is beneficial in reducing NEEs.

**Implementing effective public policies and strategies**. In response to the increasing knowledge of the implications of nanoparticles within NEEs, there is a need to formulate effective public policies and strategies. This could include developing mitigation measures, setting standards for NEEs, formulating emissions policies, and enacting relevant laws and regulations.

## CRediT authorship contribution statement

**Yingyue Wei:** Writing – review & editing, Writing – original draft, Validation, Methodology, Investigation, Formal analysis, Conceptualization. **Prashant Kumar:** Writing – review & editing, Writing – original draft, Supervision, Resources, Project administration, Methodology, Funding acquisition, Conceptualization.

## Declaration of competing interest

The authors declare that they have no known competing financial interests or personal relationships that could have appeared to influence the work reported in this paper.
